# 2024 international conference on neuroprotective agents conference proceedings

**DOI:** 10.3389/ebm.2025.10699

**Published:** 2025-07-21

**Authors:** William Slikker, Tucker A. Patterson, Slobodan M. Todorovic, Russell J. Andrews

**Affiliations:** ^1^ Retired, Little Rock, AR, United States; ^2^ National Center for Toxicological Research, Jefferson, AR, United States; ^3^ University of Colorado School of Medicine, Aurora, CO, United States; ^4^ World Federation of Neurosurgical Societies, Los Gatos, CA, United States

**Keywords:** neuroprotective agents, neurochemistry, nervous system, neuroprotection, neuropharmacology

The purpose of the International Conference on Neuroprotective Agents (ICNA) is to assimilate basic science researchers and clinicians from many countries and disciplines in a common forum to address various approaches and advancements to achieve neuroprotection. The scientific format provides the opportunity for clinicians and basic researchers to come together in a relaxed and informal manner in order to foster the free exchange of ideas and concepts among the participants. Almost all the attendees provide an oral or a poster presentation, and informal discussions are scheduled for each session. The conferences are routinely held in small venues to encourage interaction among the participants outside of the formal sessions. Meals and coffee breaks are provided so discussions can continue throughout the day and evening. Receptions and excursions provide additional opportunities for scientific exchange. The ICNA is generally held every 2 years, and all proceedings have been published except for the very first conference held in Rockland, ME, USA, in 1991 [1]. The focus of the content in this special issue demonstrates the breadth of topics that are typical of the ICNA.

In keeping with its global aspect, the 16th ICNA was held in Bentonville, Arkansas, a small but growing city in the Northwest corner of the state. Student participation was excellent, thanks to the extensive neuroscience research being conducted throughout the state of Arkansas and elsewhere. Students at the graduate or postdoctoral level were eligible to compete in a scientific platform presentation session and competitive travel fellowships were graciously provided to Arkansas University students by the Arkansas Research Alliance.

The format was similar to previous ICNAs: a Monday evening reception, followed by a full day of presentations on Tuesday plus half-day morning presentations on Wednesday and Thursday. A brief walking tour of the historic downtown Bentonville preceded a tour and dinner on Tuesday afternoon/evening at the beautiful and informative Crystal Bridges Museum of American Art.

Contents and summaries:

[2] *The effects of cannabidiol and its main metabolites on human neural stem cells*. Many pregnant women have been using CBD to treat pregnancy symptoms, causing fetal exposure to CBD. Using human neural stem cells in culture, the authors reported that CBD and its major metabolites 7-OH-CBD and 7-COOH-CBD reduced human NSC viability at concentrations comparable to those in human blood. In addition, CBD was observed to reduce GFAP and cannabinoid receptor 2 (CB2) expression after NSCs differentiation.

[3] *Anesthesia-induced developmental neurotoxicity in the setting of systemic inflammation: the role of microglia.* Sevoflurane neurotoxicity is enhanced in the setting of systemic inflammation induced by either LPS injection or trauma (tibia fracture) in terms of its onset, the intensity and duration that could, at least in part, be explained by a complex interplay between microglia activation and T-cell infiltration. Specifically, our mechanistic studies suggest that sevoflurane induced neuroapoptosis triggers activation of microglia, which in turn leads to the upregulation of proinflammatory cytokine MCP-1 and endothelial cell adhesion molecule, ICAM-1 mRNA levels in the hippocampus. This results in T-lymphocyte infiltration in the hippocampal subiculum, an event that further perpetuates microglial activation to control neuroapoptosis which is suggested by the fact that microglia depletion leads to a significant worsening of sevoflurane-induced developmental neuroapoptosis.

[4] *Assessing Potential Desflurane-induced Neurotoxicity Using Nonhuman Primate Neural Stem Cell Models.* Our data suggests that at the clinically relevant concentration, desflurane did not induce Neural Stem Cell *(*NSC) damage/death, but impaired the differentiated neuronal cells after prolonged exposure. These findings should be helpful/useful for the understanding of the diverse effects of desflurane exposure on the developing brain and could be used to optimize the usage of these agents in the pediatric setting.

[5] *Assessing the Developmental Effects of Fentanyl (Anesthetics/Analgesics) and Impacts on Lipidomic Profiling Using Neural Stem Cell Models.* These data indicated that micro molar concentrations of fentanyl exposure (24-h) did not induce detectable cell death. However, a lipidomic analysis indicated that fentanyl may affect immature neural cell functions through modifying lipid composition and lipid metabolism. These data indicated that despite the absence of clear neurodegeneration, fentanyl may still have a negative impact on the developing brain.

[6] *AlphaLinolenic acid-induced facilitation of GABAergic synaptic transmission is mediated via acid-sensing ion channel (ASIC1a) activity in the basolateral amygdala.* The selective vulnerability of inhibitory neurons may contribute to the desynchronization of oscillations and lead to hyperexcitability disorders. By enhancing inhibitory neuronal transmission and bursting, alpha-linolenic acid may restore the synchronization of oscillations to prevent epilepsy and other hyperexcitability disorders.

[7] *In vivo silencing of the thalamic Ca*_*V*_ *3.1 voltage-gated calcium channels demonstrates their region-specific role in anesthetic mediated hypnosis.* The authors silenced the *Cacna1g* gene that encodes the low-threshold-activated Ca_V_3.1 T-type voltage-gated calcium channel subunit by injecting short-hairpin RNA (shRNA) into midline and intralaminar - nonspecific thalamus (MIT) and sensory - specific ventrobasal (VB) thalamic nuclei in wild-type mice. They found that knocking down Ca_V_3.1 channels in MIT significantly decreased inhaled isoflurane concentration that is required to induce hypnosis, but it did not affect speed of anesthetic induction and the immobilizing effect of isoflurane. In contrast, knocking down the Ca_V_3.1 channel in the VB thalamus did not affect any of the measured anesthetic endpoints. Hence, they concluded that Ca_V_3.1 channels in nonspecific MIT thalamus have a preferential role in anesthetic hypnosis when compared to the sensory VB thalamus.

[8] *Comparative electrophysiological study of neuroactive steroid-induced hypnosis in mice: sex and drug-specific differences.* This study investigated sex-specific effects of two common neuroactive steroids such as alphaxalone and allopregnanolone on thalamocortical (TC) oscillations in mice that are associated with their hypnotic/sedative effects. They found that that females were more sensitive to both agents as evidenced by longer duration of hypnosis following intra-peritoneal injections of a dose of 100 mg/kg. Both agents had distinct electrophysiological signatures in TC circuitry that may underly their sedative/hypnotic effects with allopregnanolone inducing more profound TC suppression in females than males. The authors conclude that potential future use of neuroactive steroids for clinical anesthesia warrants consideration of their sex-specific effects.

[9] *Cystamine reduces neurodegeneration and epileptogenesis following soman-induced status epilepticus in rats*. Research efforts are continually investigating and aiming to improve therapies that may help alleviate neurodegeneration caused by injuries, toxic exposures, and/or neurological disorders. We present findings on the potential novel use of aminothiols as neuroprotectors, using a preclinical model of cholinergic-induced toxicity and associated neurodegeneration, highlighting the promising ability of aminothiols to reduce neuropathology when used as an adjunct to the current standard of care.

[10] *Involvement of EGFR-AKT signaling in hemin-induced neurotoxicity*. Using afatinib as a positive control, epithelial growth factor receptor (EFGR)-protein kinase B (aka AKT) signaling is implicated in hemin-induced neurotoxicity and may represent a druggable target for intracerebral hemorrhage.

[11] *A double-edged effect of hypoxia on astrocyte-derived exosome releases.* Using hypoxia-preconditioned donor cells, exosome functionality appears to have both beneficial and detrimental effects on neurotoxicity, suggesting that hypoxia preconditioning plays a double-edged role.

[12] *Limitations to Clinically Restoring Meaningful Peripheral Nerve Function Across Gaps and Overcoming Them.* This review examines the efficacies, mechanisms of action, and limitations of many of the techniques used to restore meaningful function to peripheral nerves following injury that destroys a length of a nerve, creating a gap. It concludes that a novel technique using a special formulation of plateletrich plasma (PRP) is the most effective technique and has few, if any, limitations.

[13] *Mechanisms, including PRP, for Reducing/Eliminating Chronic Neuropathic Pain.* This review examines the efficacies, mechanisms of action, and limitations of many of the techniques used to reduce neuropathic pain following peripheral nerve trauma. It concludes that a novel technique using a special formulation of plateletrich plasma (PRP) is the most effective and can not only induce long-term chronic neuropathic pain reduction but long-term pain elimination and has few, if any, limitations.
